# An Observational Cohort Study Evaluating Antimicrobial Use in Peripartum Sepsis: A Tendency towards Overdiagnosis?

**DOI:** 10.3390/pharmacy8040211

**Published:** 2020-11-11

**Authors:** Nouf Abutheraa, June Grant, Alexander B. Mullen

**Affiliations:** 1 Strathclyde Institute of Pharmacy and Biomedical Science, Faculty of Science, University of Strathclyde, 161 Cathedral Street, Glasgow G4 0RE, UK; a.mullen@strath.ac.uk; 2 Obstetrics & Gynaecology, Women & Children’s Services, NHS Greater Glasgow & Clyde, Princess Royal Maternity, 16 Alexandra Parade, Glasgow G31 2ER, UK; June.grant@ggc.scot.nhs.uk

**Keywords:** antimicrobial, antimicrobial stewardship, maternity, peripartum, pregnancy and labour, sepsis

## Abstract

(1) Background: Sepsis is the leading cause of maternal death in 11–15% of women worldwide. This emphasises the importance of administrating timely and appropriate antibiotic therapy to women with sepsis. We aimed to evaluate the appropriateness of antimicrobial prescribing in women diagnosed with peripartum sepsis. (2) Method: A prospective observational cohort study in a single Scottish health region with 12,233 annual live births. Data were collected on women diagnosed with sepsis in the peripartum period using physical and electronic medical records, drug Kardex^®^ (medication administration) and ward handover records. (3) Results: A sepsis diagnosis was concluded in 89 of the 2690 pregnancy cases reviewed, with a median hospital stay of four days. Good overall adherence to the local guidelines for the empiric antimicrobial treatment of sepsis was observed. Group B Streptococcus was associated with 20.8% of maternal sepsis cases, whilst in 60% of clinical specimens tested no causative pathogen was isolated. (4) Conclusion: The lack of specific and sensitive clinical markers for sepsis, coupled with their inconsistent clinical application to inform diagnosis, hindered effective antimicrobial stewardship. This was further exacerbated by the lack of positive culture isolates from clinical specimens, which meant that patients were often continued on broader-spectrum empiric treatment.

## 1. Introduction

Sepsis is the presence of infection in conjunction with the systemic inflammatory response syndrome (SIRS) with or without organ dysfunction, and was defined in 2016 as a “life-threatening organ dysfunction caused by a dysregulated host response to infection” [1], which can be measured using the sepsis-related organ failure assessment (SOFA) or the quick-SOFA (qSOFA) [1]. Sepsis is the leading cause of maternal death in 11–15% of women worldwide [2,3]. In the UK and Ireland between 2009 and 2012, it accounted for approximately 2–2.5 maternal deaths in every 100,000 pregnancies [4,5]. Sepsis is the second most common cause of obstetric admission to critical care units [6], responsible for up to 22% of intensive care unit (ICU) admissions in the maternity population [7]. One woman in every 1000 pregnancies experiences sepsis, and the incidence of sepsis during pregnancy or the postpartum period has been reported to be 1–3% [8,9].

The physiological changes associated with an obstetric population, especially within the third trimester, increase the diagnostic challenge of applying abnormal SIRS parameters as a sign of infection [10]. A confirmed microbial infection takes at least 24 h and it only occurs in 30–40% of severe sepsis patients, which adds to the challenge of diagnosing sepsis [11]. In these circumstances, when sepsis is suspected, antimicrobial intervention is delivered as quickly as possible following the provisional diagnosis on the basis of expediency [2]. The administration of intravenous broad-spectrum antimicrobial to women with suspected or confirmed peripartum sepsis is recommended [10,12,13,14]. Care should be considered and delivered within a one-hour window as recommended by the Royal College of Obstetricians and Gynaecologists (RCOG) and the Surviving Sepsis Campaign [12,14]. The choice of antimicrobial should follow local guidelines and policy, which are mainly based on the epidemiology of the particular hospital and/or geographical area [12,15]. However, the antimicrobial stewardship strategy in the management of sepsis cases is to create a balance between reducing antimicrobial resistance and managing the suspected infection [16]. The de-escalation process is a fundamental protocol of antimicrobial stewardship and encourages cessation when antimicrobial therapy is started in non-infection cases [16]. Therefore, initiating intravenous broad-spectrum antimicrobial treatment should prompt daily clinical review and cessation or de-escalation to a narrower-spectrum antimicrobial and/or intravenous to oral switching when appropriate [13,14]. Evidence suggests that there is a reluctance to change antimicrobial therapy in sepsis cases due to the unknown safety and efficacy of this behaviour, and this overlooks the fact that antimicrobial resistance and sepsis are “two sides of the same coin” [16].

Antimicrobial stewardship aims to reduce unnecessary antibiotic exposure and prompt appropriate antibiotic use across all settings [17]. In maternity, it has been integrated within its practice in various ways. In preterm pre-labour rupture of the membrane, the recommendation now is to receive only 72 h of IV antibiotic therapy, as evidence has revealed that Group B *streptococcus* (GBS) cannot be isolated from the women after three days of treatment [18]. Penicillin allergy testing has also been recommended to women with positive GBS to exclude cases of untrue penicillin allergy as part of antimicrobial stewardship goals [18]. However, these initiatives were found to be limited in sepsis management, and there was a concern of overprescribing behaviour of antibiotic therapy in the management of sepsis in maternity wards. This study aims to quantify the types of antibiotic agents prescribed and evaluate their appropriateness in women diagnosed with peripartum sepsis., observe the clinical parameters used to assist the diagnosis of peripartum sepsis and describe bacterial isolates in patients with suspected or confirmed sepsis.

## 2. Materials and Methods

A prospective observational cohort study was conducted in three maternity hospitals of a single Scottish health region that recorded 12,233 live births. This observational cohort study undertaken was the initial phase of a quality improvement programme to improve alignment between the clinical governance and management of peripartum sepsis and antimicrobial stewardship in an obstetric setting. Data were collected over a 12 week period to include women diagnosed with suspected or confirmed sepsis. We excluded women based upon age (≤16 years of age; n = 0) or where antibiotics were prescribed prophylactically.

Sepsis cases were identified using ward handover sheets and patients’ handwritten medical notes. Data were collected from individual medical notes, drug Kardex^®^ (medication administration) records and electronic records. For one patient, the drug Kardex was not accessible; therefore, the results section reports antibiotic therapy prescribed to 88 of the 89 identified patients.

Individual patient demographics including age, body mass index (BMI), parity, gravidity, child gestational age at delivery and duration of hospital stay were collected. Data on mode of delivery were collected, except for antenatal sepsis (n = 7) and for some postnatal sepsis readmissions, when medical notes were not available in the ward upon readmission (n = 4). SIRS criteria were used by the healthcare professional during the study period to calculate individual patient scores based on body temperature, heart rate, respiratory rate and white cell count values, which were collected for each woman near the time of diagnosis (Table 1).

Data were also collected from individual patient microbiology reports indicating whether a pathogen was isolated/identified from the culture or swab specimens.

Antibiotic treatments prescribed (type, dose, route and duration) were collected from each patient’s drug Kardex and the subsequent immediate discharge letter (IDL), a document containing a summary listing of the patient’s medication at discharge. From this information, we calculated the number of antibiotic therapies for each patient, with any changes in the dose or route of administration being considered to constitute a new therapy. Also, antibiotic appropriateness was assessed based on the local hospital guidelines.

The accuracy, completeness and consistency of database entries were validated by co-author JG. Individual patient data were immediately anonymised and held electronically on a secure National Health Service (NHS) server until analysis was performed. All analysis was performed using the IBM SPSS Statistics for Windows, version 23.0 (IBM Corp., Armonk, N.Y., USA) using a significance level of 5% for data interpretation.

### Definitions

- **Sepsis diagnosis:** the clinical diagnosis of sepsis according to the written information within a patient’s medical notes and not limited to a positive microbiology report or a patient’s SIRS score.
- **Suspected sepsis:** women who were diagnosed with sepsis and started on empiric antibiotic therapy regardless of their medical (i.e., their SIRS score) or microbiology status.
- **Confirmed sepsis:** women who were diagnosed with sepsis and started on empiric antibiotic therapy and have a positive culture.
- **Postnatal sepsis**: sepsis suspected or diagnosed at least 24 h after delivery, including all readmissions reported during the data collection period and within 42 days postpartum.

## 3. Results

A total of 89 patients with a mean age of 29.8 ± 5.3 years were identified with suspected or confirmed sepsis from a total of 2690 pregnancies. This gave an incidence rate of 3.3%. In women with suspected or confirmed sepsis, 46.1% (n = 41) were delivered by emergency caesarean section, 1.1% (n = 1) by elective caesarean section, 24.7% (n = 22) by spontaneous vaginal delivery and 15.7% (n = 14) by instrumental delivery, while data were missing for 13.5% (n = 11) of women. Most cases (85.4%, n = 76) of sepsis diagnosed during hospital admission were reported in the labour (46.1%, n = 41) and postnatal (39.3%, n = 35) wards. In 89.9% (n = 80) of cases women were treated in maternity wards, while 7.9% (n = 7) of the women were treated in high dependency units (HDUs) and 2.2% (n = 2) were treated in an intensive care unit.

Based on antenatal booking data (i.e., the woman’s first appointment with the midwife, which is normally between week 8 and 12 of pregnancy), no women were underweight. Twenty-two (27.8%) women were obese (45.5% were class I obese (BMI 30–34.9 kg/m^2^); 27.3% were class II obese (BMI 35–39.9 kg/m^2^) and 27.3% were class III obese, with BMI ≥ 40 kg/m^2^). Additional patient demographic data are summarised in Table 2.

**Figure 1:**
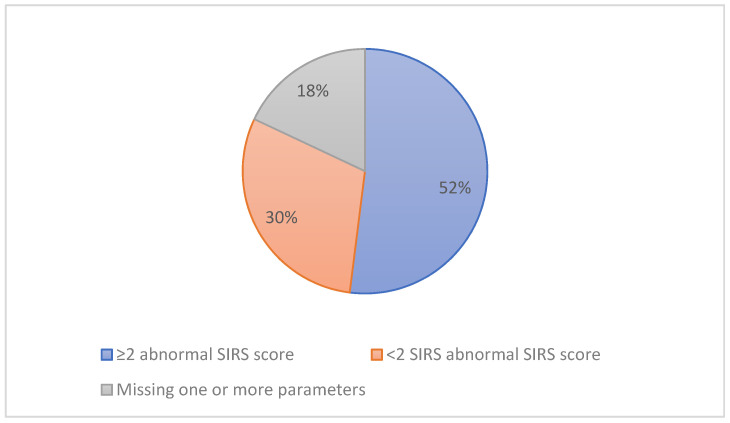
The percentage of women diagnosed with suspected or confirmed sepsis and their SIRS score.

We evaluated the SIRS score for the 89 women and found that only 46 patients scored two or more, 27 patients scored less than two and 16 patients had one or more parameters missing at the point of sepsis diagnosis (Figure 1). In cases where SIRS scored less than one, physicians were diagnosing sepsis in these women based on their physical examination and clinical experience.

### 3.1. Antimicrobial Therapy

Table 3 summarises antimicrobial therapy administered, ranging from 1 to 17 therapies per patient, with a median of 3 antibiotic therapies per patient. Duration of therapy varied between hospital admission and discharge, having a median of 2 (1–5 days) for antibiotics prescribed in the ward and 7 (3–14 days) for IDL antibiotic prescriptions.

The IDLs of all 89 patients were assessed, revealing that 18 women (20.2%) were discharged without antimicrobial therapy. Six of the 18 patients had been considered septic based only on the clinical criteria of “pyrexia in labour”, and antimicrobial therapy was subsequently discontinued after 24 h. There were no documented reasons for discontinuation of antimicrobial therapy for the remainder. Among the 18 patients, only two had a positive culture. Upon further investigation we found that one patient was discharged after 31 days and completed her antibiotic therapy during her stay and that the other patient had coliform bacteria in her urine culture.

Patients’ IDLs revealed that 61.8% (n = 55) of the women were discharged on 625 mg of oral amoxicillin/clavulanic acid three times daily (TDS). This included many patients where no pathogen was isolated and who were initiated on 1200 mg intravenous amoxicillin/clavulanic acid TDS.

### 3.2. Microbiology Reporting

**Figure 2:**
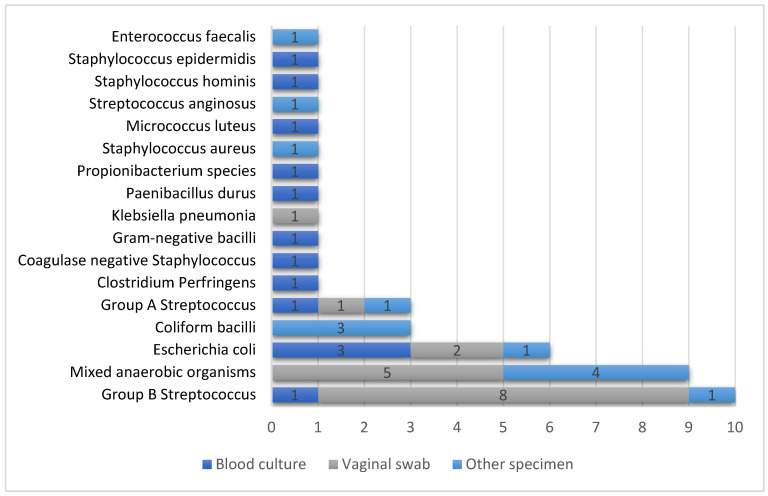
Pathogens isolated from the positive culture results of women diagnosed with sepsis.

A total of 120 blood cultures or swabs from the 89 patients were analysed. The microbiology lab reported no cultures processed for four patients. The majority of specimens showed “no growth” of a pathogen (60%; n = 72). Figure 2 summarises the results of positive culture or swab based on specimens (n = 48, 40%). One case each of *Clostridium perfringens*- and *Escherichia coli*-associated sepsis (n = 2) were associated with ICU admission and prolonged hospital stay of 30–31 days duration.

**Figure 3:**
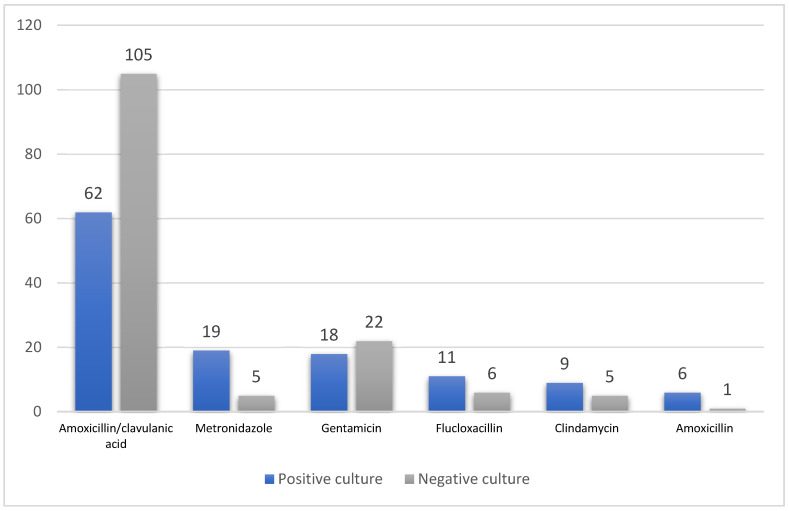
The most common antibiotic prescribed for culture-positive and culture-negative patients.

The number of antibiotic prescriptions ranged from 1 to 17 therapies in patients with culture-positive results and from 1 to 7 therapies in patients with culture-negative results. Amoxicillin/clavulanic acid was the most commonly prescribed therapy in 105 and 62 cases of negative and positive cultures, respectively (Figure 3). Additional antibiotic therapies were prescribed for only positive culture patients (Table 4). No significant differences were found in weight, BMI, parity, gravidity, or estimated blood loss between culture-positive and culture-negative patients. However, a culture-positive patient had a significant longer length of hospital stay; *P* = 0.007.

## 4. Discussion

The apparent incidence of sepsis was 331 in every 10,000 pregnancies. This appears high relative to the literature, which reports the incidence of sepsis as 10 in every 10,000 pregnancies [19]. However, our study included all suspected or confirmed cases that were treated with antibiotics, whereas Acosta et al. report only confirmed cases from a retrospective cohort study and did not include cases where antibiotic therapy may have been initiated for suspected sepsis [19]. In this study, sepsis was mainly diagnosed within the intra-partum (46.1%) and postpartum (39.3%) periods. This is in agreement with a recent study from the Republic of Ireland which reported figures of 36% and 47.1% respectively for intrapartum and postpartum sepsis [8]. The median length of stay in hospital was four days (IQR: 3–6 days), similar to a recent US study of maternal sepsis [19]. In comparison, the reported length of stay for a general obstetric population is reported to be 3 days (IQR: 2–4 days) [20]. The proportion of patients admitted to an HDU or an ICU was 7.9% and 2.2% respectively, slightly lower than those reported in the Irish study [8]. Obesity was observed in 27.8% of our sepsis cohort, slightly higher than the 22.2% of women who gave birth in Scotland with a BMI >30 kg/m^2^ [21]. Obesity is known to increase the risk of developing infection 3.5-fold when compared with non-obese women [3]. An emergency caesarean section was observed in 46.1% (n = 41) of septic women, which is similar to other studies and is associated with a 20-fold greater risk of developing infection than for spontaneous vaginal delivery [3].

The presence of at least two abnormal SIRS criteria is a factor aiding the clinical identification of sepsis. Nonetheless, only 51.7% of the patient cohort met these criteria, with 30.4% exhibiting none or only one abnormal SIRS criterion. Pyrexia in labour was the leading single clinical reason to initiate antibiotic therapy in this study cohort, despite 30% of women known to experience intrapartum pyrexia [22]. Pyrexia can be attributable to various sources of infection, including chorioamnionitis, but may also arise from epidural analgesia [22]. Elevation in temperature is accompanied by many other maternal changes at delivery, including tachycardia and altered respiratory rate, which in most cases normalise quickly [22]. This may explain the proportion of patients where cessation of antibiotic therapy occurred within 24 h post-delivery with reversion to normal physiological status.

The overuse of antimicrobials has been reported worldwide within obstetric populations, with a recent study of antimicrobial use in postpartum women finding that only 7% of total antibiotic use followed a confirmed diagnosis of infection [23]. There was a high proportion of adherence to local guidelines, where intravenous amoxicillin/clavulanic acid with or without gentamicin as first-line therapy for suspected sepsis is recommended, with clindamycin the preferred substitution for amoxicillin/clavulanic acid in penicillin-allergic women [24]. This was observed in our cohort study as patients were treated empirically with these medications. It is important to highlight that quick action is required to treat sepsis cases, but de-escalation is necessary because the inappropriate use of antimicrobial agents is one of the main drivers for developing antimicrobial resistance [25]. It has been reported that sustained high-volume antibiotic usage has meant that extended-spectrum beta-lactamases are becoming increasingly common in the obstetric population [26]. The de-escalation process was limited in patients with a negative culture, and evidence suggests that clinical review of antibiotic prescriptions after the third day is shown to reduce antibiotic duration of therapy, compared with usual care, *P* < 0.001 [27]. Leone and colleagues found no significant difference in the discharge rate, duration of antibiotic treatment and 90 day mortality before and after the de-escalation protocol was implemented in the ICU, *P* = 0.7, *P* = 0.94 and *P* = 0.35, respectively [28], which emphasised the safety of the de-escalation protocol and encourages healthcare professionals to de-escalate when possible to slow the development of antimicrobial resistance.

In our study, patients with a positive culture received more antibiotic therapy and experienced a longer stay in hospital. Broad-spectrum antibiotics like vancomycin, meropenem, aztreonam, piperacillin/tazobactam and others were used in a very limited number of patients. This represents a high awareness of the management of sepsis and antimicrobial stewardship awareness as these antibiotics were not used in culture-negative patients but were only considered in critically ill cases. An association was found in positive culture sepsis between inadequate antimicrobial therapy and hospital death (adjusted OR: 1.40; 95%CI, 1.07–1.84; *P* = 0.02) [29]. Therefore, it is important to provide adequate antimicrobial coverage when treating sepsis women with a positive culture, which was observed in the current study. The main pathogens isolated were Group B *streptococci*, mixed anaerobic organisms, *E. coli*, *coliform* bacteria, and Group A *streptococci*. Previous studies recorded comparable findings [8]. *E. coli* is the most common pathogen associated with maternal sepsis [9] and is reported to be associated with up to 31% of genital tract-related sepsis cases in the obstetric population [30]. *S. pyogenes* and *E. coli* are common pathogens in clinical cases of chorioamnionitis, and combinations of Gram-positive and Gram-negative bacteria are common in maternal sepsis, according to the RCOG [12]. *Coliform* bacteria have been reported in cases of urinary sepsis, while *Clostridium perfringens* is a less common pathogen [12]. Although 60% of microbiology specimens did not isolate a pathogen, this did not negate the clinical diagnosis of sepsis, as antibiotic therapy should not be discontinued on the sole basis of a negative microbiology report [31]. Meanwhile, others suggest that as long as no evidence of infection can be revealed, it is recommended to stop the empiric therapy, and evidence supports the safety of such an approach [32]. Different tools and strategies have been implemented to encourage prescribers to consider antibiotic de-escalation, including 72 h time-outs [27,32]. This 3 day review period provides sufficient time to gather diagnostic evidence, make a clinical assessment and monitor the patient’s condition to ensure that the right decision is made for each patient. An education initiative about the de-escalation protocol and the 72 h time-out could be initiated as a mandatory follow-up for all patients started on empiric therapy. Meanwhile, patients with a negative culture could have this review before hospital discharge when the 72 h is longer than their hospital stay.

This study had several limitations. Due to finite resources, data were only collected over the defined period. The findings reported in this study may also not be generalizable to all maternity wards, but it accurately captured the current clinical practice within a single health authority.

## 5. Conclusions

Accurate early diagnosis of sepsis is critical so that appropriate treatment can be promptly initiated to provide patients with the best possible chances of survival. The clinical challenge of identifying sepsis in an obstetric population is complex and exacerbated by the physiological changes associated with pregnancy and inflammatory processes associated with birth methods [10,33]. However, negative microbial cultures should not hinder the possibility of antimicrobial de-escalation or therapy cessation. Although it is indisputable that appropriate use of antibiotics in the treatment of true sepsis is lifesaving, the potentially negative impact of unnecessary antibiotics on maternal and fetoplacental microbiomes is becoming apparent, reinforcing the need for judicious use of these agents wherever possible [34,35].

The management of maternal sepsis with positive microbial cultures shows an escalation of therapy from amoxicillin/clavulanic acid with or without gentamicin to meropenem, aztreonam, piperacillin/tazobactam or vancomycin in some critically ill patients. These therapies were only observed in a very small cohort of this study and were justified and reflected good clinical practice. The antimicrobial stewardship protocol should be empowered, particularly in dealing with culture-negative patients to encourage the de-escalation or cessation of unnecessary antibiotic therapy. Further research is, therefore, required to empower antimicrobial stewardship within obstetric settings, with integration of other healthcare professionals such as midwives and pharmacists in this process being fundamental to its success.

## Figures and Tables

**Table 1:** Systemic inflammatory response syndrome (SIRS) criteria according to the local guidelines.

	Modified SIRS Criteria for Maternity Wards
Temperature	<36 °C or >38 °C
HR ^1^	>100 beats per minute
WCC ^2^	<4 or >16 × 10^9^/L
RR ^3^	>20 breaths per minute
SBP ^4^	<90 mmHg
Mental status	Altered mental status

^1^ HR: Heart Rate ^2^ WCC: White cell count; ^3^ RR: Respiratory rate; ^4^ SBP: Systolic blood pressure.

**Table 2:** Patients’ demographic data (n = 89).

Demographic/Maternal Data	Median (Range)
Weight (kg)	70 (44–154)
Body Mass Index (kg/m^2^)	25.3 (18.7–55.1)
Parity	0 (0–8)
Gravidity	0 (0–10)
Gestation age (week)	39.5 (13–41)
Length of hospital stay (day)	4 (1–31)
Estimated blood loss (ml)	900 (100–5000)

**Table 3:** The number of prescriptions administered to suspected/confirmed septic women.

	Number of Prescriptions (%)			
**Antibiotic name/class ^1^**	Total (100%)	SIRS ≥ 2 (59.4%)	SIRS < 2 (28.8%)	Unknown (11.8%)
Penicillins	213 (68.05%)	124 (66.7%)	59 (65.5%)	30 (81.1%)
Cephalosporin, Carbapenems and other beta-lactams	7 (2.24%)	7 (3.7%)	0	0
Aminoglycosides	40 (12.78%)	24 (12.9%)	12 (13.3%)	4 (10.8%)
Macrolides	5 (1.60%)	2 (1.1%)	3 (3.3%)	0
Clindamycin	14 (4.47%)	10 (5.4%)	2 (2.2%)	2 (5.4%)
Vancomycin	5 (1.60%)	2 (1.1%)	3 (3.3%)	0
Trimethoprim	2 (0.64%)	2 (1.1%)	0	0
Metronidazole	25 (7.99%)	13 (6.9%)	11 (12.2%)	1 (2.7%)
Quinolones	2 (0.64%)	2 (1.1%)	0	0
**Total**	313	186	90	37

^1^ Based on the British National Formulary (BNF) categories.

**Table 4:** Additional antibiotic therapies that were prescribed only to positive culture patients.

Antibiotic Name	Number of Prescriptions
Vancomycin	5
Benzylpenicillin	4
Aztreonam	3
Clarithromycin	3
Piperacillin/tazobactam	3
Ciprofloxacin	2
Erythromycin	2
Meropenem	2
Temocillin	2
Trimethoprim	1
Cefalexin	1
Ceftriaxone	1
